# Energy Supplementation during the Last Third of Gestation Improves Mother–Young Bonding in Goats

**DOI:** 10.3390/ani11020287

**Published:** 2021-01-23

**Authors:** Juan M. Vázquez-García, Gregorio Álvarez-Fuentes, Héctor O. Orozco-Gregorio, Juan C. García-López, Milagros González-Hernández, César A. Rosales-Nieto

**Affiliations:** 1 Facultad de Agronomía y Veterinaria, Universidad Autónoma San Luis Potosí, San Luis Potosí 78321, Mexico; manuelvazquez87@yahoo.com.mx (J.M.V.-G.); gohector72@yahoo.com.mx (H.O.O.-G.); milagros.gonzales@uaslp.mx (M.G.-H.); cesar.rosales@uaslp.mx (C.A.R.-N.); 2 Asociación Mexicana de Criadores de Ganado Caprino de Registro, San Luis Potosí 78433, Mexico; 3 Instituto de Investigaciones de Zonas Desérticas, Universidad Autónoma de San Luis Potosí, San Luis Potosí 78377, Mexico; jcgarcia@uaslp.mx

**Keywords:** goats, gestation, energy supplementation, maternal behavior, neonatal behavior

## Abstract

**Simple Summary:**

The last third of gestation is a period of high energy demand for the dam, because she needs to support the growth of fetuses and the newborn. Moreover, towards the end of gestation, maternal feed intake is reduced as the mass of the fetoplacental unit increases. The maternal diet often cannot meet nutritional requirements, compromising thermoregulatory capacity, wellbeing, and viability, and the survival of the newborn. We have shown that energy supplementation of the mother during the last third of gestation does not affect progeny birth weight but enhances mother–young bonding.

**Abstract:**

We tested whether maternal energy supplementation during the last third of gestation improves birth weight, neonatal wellbeing, and mother–young bonding. Thirty-six pregnant French Alpine goats were randomly allocated among three nutritional treatments for the last third of pregnancy: (i) Control, fed alfalfa (T-0; *n* = 12); (ii) alfalfa + 150 g/head daily energy concentrate (T-150; *n* = 12); (iii) alfalfa + 300 g/head daily energy concentrate (T-300; *n* = 12). At birth, we collected progeny data on birth weight, birth type, sex, rectal temperature, heart rate, respiratory rate, time to standing, time to udder connection, and time to first feeding. For the dams, we collected data on the duration of labor, time to clean the progeny, and time to allow first suckling. At birth, body weight, rectal temperature, heart rate, and the respiratory rate did not differ among treatments (*p* > 0.05). In the dams, labor duration was not affected by the treatments (*p* > 0.05). The T-150 dams were faster to clean the newborn and allow first suckling (*p* < 0.05). The T-150 progeny were faster to stand and the T-300 progeny were faster to connect to the udder (*p* < 0.05). We conclude that energy supplementation of the dam during the last third of gestation does not affect the birth weight of the progeny, but enhances the mother–young bonding.

## 1. Introduction

Goats are distributed across diverse geographical and agro-ecological zones around the world, with nearly 95% of them found in developing countries [1]. Importantly, goats are used for the production of milk and meat, and are usually the principal source of income in low-input farming systems [2]. Mexico, for example, has almost 9 million goats, mostly in arid and semi-arid conditions, that contributed to the economy in 2019 by producing nearly 40,000 tons of meat [3]. Although they are very resilient animals, their fertility rate can be reduced to 70% when the nutritional requirements are not met, with females that conceive losing fetuses, resulting in a kidding rate of about 50%. This is a significant economic loss and animal welfare issue for the industry [4,5]. The combination of undernutrition and poor health leads to low reproductive efficiency and genetic pressure on survival rather than productivity.

With respect to nutrition, the last third of gestation and early lactation are of interest because they are periods of high maternal energy demand to support the growth of fetuses and the newborn [6,7]. Around 75% of fetal growth occurs in this period and, to make matters worse, feed intake is reduced towards the end of gestation because the growth of the fetoplacental unit restricts rumen capacity [8,9]. When the nutritional requirements are not met, the transfer of energy, protein, and essential nutrients from the mother to the fetus are reduced, compromising fetal growth and development [8,10]. Ultimately, the fetus can adapt to these nutritional challenges by modulating growth, but birth weight is reduced [11,12,13]. Moreover, nutritional constraints disrupt metabolic endocrine activity in the dam, leading to a catabolic state [14,15] that is associated with inadequate mammary gland development and prolonged labor. The outcome for the neonate is a poor ability to stand and suck, coupled with low quantity and quality of colostrum and milk—a situation magnified as the number of fetuses increases [16,17,18,19]. Importantly, colostrum and milk yield is correlated with mother–young bonding at birth, neonatal survival, and postnatal growth [20,21,22,23].

From birth, the newborn quickly adapts to the extra-uterine environment by modifying its homeothermy mechanisms—core body temperature, heart rate, and respiration rate [24,25]. Despite these adaptational processes, most neonatal mortality occurs from birth to 72 h post-partum, with the greatest proportion seen in twin births and larger litters [26]. Immediately after birth, the mother and the neonate establish a strong and selective bond that is initially mediated by olfactory, auditory, and visual signals, and then reinforced during early lactation by the consumption of colostrum and milk [27]. Nevertheless, poor mother–young bonding and perinatal kid loss are strongly associated with low birth weight and mismothering [28]. Importantly, maternal undernutrition during gestation can compromise fetal thermoregulatory capacity and, consequently, reduce the wellbeing and viability of the neonate [29,30,31]. Moreover, in addition to the effects of low neonatal weight and wellbeing on mortality [26,32], there will be a reduction in postnatal growth that can have life-long effects on reproductive efficiency [33,34,35].

Considering this situation, it is clearly necessary to develop simple, low-cost strategies that will allow goat farmers to increase the productivity and reproductive efficiency of their animals. Such strategies will precisely coordinate nutritional inputs with the stages of the reproductive process to ensure that the metabolic signals are appropriate for optimizing the wellbeing and vitality of the neonate. Energy supplementation two weeks before parturition has been reported to increase colostrum quality and neonatal survival [36,37], but it is not known whether the benefits for the newborn are due to improvements in its wellbeing, vigor, or postnatal growth. We, therefore, tested whether gestational energy supplementation during the last third of pregnancy would improve birth weight, neonatal wellbeing, and mother–young bonding in goats.

## 2. Materials and Methods

### 2.1. Ethics Statement

This study was conducted during the breeding period at the Caprino Genetic Improvement Center in northern Mexico (22°15′ N, 100°52′ W). All procedures were consistent with the International [38] and National [39] Research Council’s Guide for the Care and Use of Laboratory Animals. The Bioethics Committee of the Faculty of Agronomy and Veterinary Medicine of the UASLP approved this experiment (reference number C19-FAI-05-0.3.03).

### 2.2. Animals and Experimental Procedure

From 55 multiparous French Alpine goats that had been naturally mated with experienced bucks for 42 days (two full reproductive cycles), 36 females on the same day of pregnancy were selected and, along with their progeny (32 females and 31 males), were used to investigate the effect of energy supplementation during the last third of gestation (from day 100 of pregnancy to delivery) on birth weight, neonatal wellbeing, and mother–young bonding. All goats were managed, including deworming and vitamin supplements, according to the standard procedures of the Genetic Improvement Center, and they had free access to clean water and a block of mineral salts containing at least 17% P, 3% Mg, 5% Ca, and 75% NaCl.

Pregnancy, number of fetuses, and gestational age were assessed three times, between 30 and 45 days after the start of mating, by transabdominal ultrasonography (Samsung-Medison SA-600 fitted to a 4 MHz convex probe; Samsung Co. Seoul, Korea). Gestational age was estimated by assessing fetometric parameters: uterine depth (in early pregnancy), fetal crown–rump length, fetal biparietal diameter, and calcification of the fetal ribs and skull [40,41,42].

### 2.3. Experimental Diets

On estimated gestational day 100 ± 4, the goats were randomly allocated among three 120 m^2^ pens, one for each dietary treatment, ensuring the average body weights of the groups were similar. The treatments were T-0 (control, receiving alfalfa ad libitum; *n* = 12; 56.8 ± 1.9 kg); T-150 (alfalfa ad libitum + 150 g/head daily energy concentrate; *n* = 12; 56.9 ± 1.7 kg) and T-300 (alfalfa ad libitum + 300 g/head daily energy concentrate; *n* = 12; 55.5 ± 1.7 kg). The nutritional content of the diet and the energy concentrate is reported in Table 1. The dry matter intake for the treatment groups was calculated based on the nutrient requirements for a 50 kg pregnant goat with low physical activity [43]. In T-150 and T-300, the energy supplement was provided daily at 07:00 h. To ensure that each goat consumed only its corresponding ration of supplement, the feeders had individual traps so the hierarchy of the dams was controlled at the time of supplementation.

### 2.4. Birth Weight and Neonatal Wellbeing

On the day of kidding, date, sex, birth type, and birth weight were recorded. An hour and a half after the end of the kidding process, the kid rectal temperature was determined (GLA model M900 thermometer, GLA Agricultural Electronics, San Luis Obispo, CA, USA.), and the respiration and heart rates were estimated with a stethoscope (CK-A601DP-07, Spirit Medical, New Taipei City, Taiwan). A total of 66 kids were born, of which 3 died in the days after birth (2 from T-0 and 1 from T-150); their data were not included in the analysis.

### 2.5. Mother–Young Bonding

Maternal and neonatal behaviors were observed by an experienced operator standing approximately 10 m away from the new mother and its progeny. This approach was backed up with a closed-circuit system in which 6 cameras were placed on the top and side of the pens, providing a panoramic view of each pen (Kit Cctv 3 Cameras HD 720p Dyr Closed Circuit, SECUCORE, Coahuila, México). The onset of parturition was detected when the behavior of the dam began to change, with the goat moving away from the others, becoming restless (lying down, getting up, walking in circles), and vocalizing. The time of the first contractions was noted as the start of labor. The following variables were then recorded for the dams: labor duration, time to clean the newborn, and delay to first suckling. The following variables were recorded for the newborn: delay from birth to standing and delay to first connection to the udder.

### 2.6. Statistical Analysis

A Shapiro–Wilks normality test was used to show that the data were normally distributed (*p* = 0.2328). Subsequently, a completely randomized design using linear mixed model procedures (PROC-MIXED) in SAS [44] was used to analyze the data. Each goat was considered as an individual experimental unit. Labor duration, time to clean the newborn, delay to first suckling, delay to standing, and time to connect with the udder, birth weight, rectal temperature, respiratory rate, and heart rate, with treatment, birth type, and progeny sex included as fixed effects in the model. For progeny variables, birth weight data were included as a covariate.

All 2-way interactions among fixed effects and covariates were included in each model, and non-significant (*p* > 0.05) interactions were removed from the model. Finally, an analysis of orthogonal polynomials was carried out to compare treatments.

## 3. Results

### 3.1. Birth Weight

Birth weight did not differ among treatments (*p* > 0.05; Table 2). Of the kids born, 49% were males and 51% were females, with the males (3.8 ± 0.7 kg) heavier at birth than the females (3.4 ± 0.5 kg; *p* < 0.01; Table 2). Of the kids born, 21% were singletons, 60% were twins, and 19% were triplets, with the singletons tending (*p* > 0.05) to be heavier (3.8 ± 0.8 kg) at birth than twins (3.7 ± 0.5 kg) or triplets (3.3 ± 0.7 kg). The difference did not differ statistically (Table 2).

### 3.2. Neonatal Wellbeing at Birth

At birth, the average rectal temperature was 38.4 °C (range 32–41 °C), the average heart rate was 188 bpm (range 136–240 bpm), and the average respiration rate was 74 bpm (range 36–180 bpm), and treatment did not affect these measures of neonatal wellbeing (*p* > 0.05; Table 2). There were also no differences between male and female kids (*p* > 0.05; Table 2), but birth type influenced heart rate and respiratory rate, with twins having lower values (*p* < 0.05; Table 2).

### 3.3. Mother–Young Bonding

#### 3.3.1. Maternal Behavior

The average labor duration was 1.6 h (range 1.2–2.4 h) and did not differ among treatments (*p* > 0.05; Table 3). The average time to clean the newborn was 34.5 min (range 15–90 min). Dams from the T-150 treatment cleaned the newborn faster after parturition (*p* < 0.001; Table 3). The average delay to first suckling was 20.5 s (range 10–42 s). Compared to control values, the delay was shorter for T-150 dams but longer for T-300 dams (*p* < 0.001; Table 3).

#### 3.3.2. Newborn Behavior

The average time taken by newborn kids to stand was 29 min (range 14–50 min), with T-150 kids being faster and T-300 kids being slower compared to control kids (*p* < 0.001; Table 3). The average time taken for newborn kids to connect with the udder was 11.5 min (range 5–20 min), with T-300 kids being faster and T-150 kids being slower, compared to control kids (*p* > 0.05; Table 3).

## 4. Discussion

Contrary to expectations, energy supplementation of multiparous female goats during the last third of gestation did not affect the birth weight of the progeny, their rectal temperature, heart rate, or respiration rate. On the other hand, mother–young bonding was improved with the T-150 supplement, as evidenced by the more rapid cleaning of the newborn and the shorter delay to standing. With respect to the delay in the first attachment to the udder, the situation was not so clear, with T-300 kids being faster and T-150 kids being slower. In general, these observations are consistent with the expected effects of maternal energy status on mother–young bonding behaviors, the thermoregulatory capacity of the newborn, and neonatal wellbeing.

The maternal diet offered during the last third of gestation did not meet energy requirements, yet the birth weight of the progeny was not significantly affected by treatment, extending on observations by Mellado et al. [45] in crossbred goats and Laporte-Broux et al. [46] in Saanen and French Alpine goats, who also found that birth weight was not affected by maternal nutritional restriction during the last third of gestation. Conversely, He et al. [47] observed that the restriction of energy or protein intake during the last third of gestation reduced the birth weight in Liuyang goats, consistent with a reduction in the development of muscle and adipose tissue in the fetuses [8,30,48]. It is not clear why the report by He et al. [47] disagrees with the others. Most observations show little effect of restricted maternal nutrition on birth weight, supporting the idea that goats are very resistant to changes in food availability, and continue to be productive because they can adapt their metabolism and physiological status when the supply of energy and protein is limited [23,49,50,51,52]. The ability of goats to adapt to adverse conditions may be programmed during fetal, rather than postnatal, development, and in response to intrauterine conditions that regulate its growth [13].

### 4.1. Neonate Wellbeing

Maternal energy supplementation during the last third of gestation did not affect the rectal temperature of the neonates. A higher rectal temperature indicates a greater thermoregulatory ability to adapt to the extra-uterine environment and is associated with increased viability and survival [53]. Interestingly, rectal temperature was higher in neonatal triplets than in singletons, the difference in rectal temperature between birth type (singleton vs. twins vs. triplets) is probably related to birth weight because triplets are born 15% lighter than singletons. Our observations extend to those by Giannetto et al. [25], in neonatal sheep and goats, that rectal temperature was inversely related to birth weight. Conversely, Dwyer and Morgan [54], and Miller et al. [55] found that neonatal rectal temperature is directly related to birth weight. As previously indicated, the difference in rectal temperature at birth could be related to the degree of maturity and birth order of the newborn; the fetus that grows fastest and thus reaches mature weight sooner is the firstborn [25,56]. It seems likely that a low birth weight leads to the activation of the neonate’s homeothermic mechanism, increasing its thermoregulatory capacity [57], perhaps due to the ability of brown adipose tissue to increase metabolism by the regulation of circulating metabolic hormones and metabolites [54,58,59,60]. Additionally, within a genotype, there is considerable genetic variation [61,62] that is likely to include rectal temperature and metabolic homeostasis at birth [54,60,63,64,65].

Glucose circulating in the mother and fetus would probably have been increased by the energy supplements fed during the last third of gestation, leading to an improvement in the physiological status of the neonate [66], but we did not observe any effect of the treatment on heart rate or respiration rate in the neonates. Heart rate should reflect homeostatic and nutritional status, and the development of the heart tissue [67], so it is seen as an indicator of physiological stress that itself is related to metabolic activation in the neonate [68]. The newborn loses a large amount of body heat through the skin in the first hours after parturition, as it adapts to its new environment, leading to a decrease in respiration rate [56,69]; therefore, it is possible to hypothesize that the newborns with lower respiration rate presented a better adaptation to the new environment [70]. Moreover, we found that newborn singletons had a higher heart rate than twins or triplets, whereas triplets had a higher respiratory rate than singletons or twins. Giannetto et al. [25] did not observe a significant difference in respiratory rate between singletons and twins, but they observed that the heart rate was higher in twins compared to singletons, attributing this event to the maturation of the newborn. Nevertheless, maternal energy supplementation during the last third of gestation had no consistent effects on the measures of neonatal wellbeing.

Many previous reports have shown that, at birth, males are heavier than females and singletons are heavier than twins or triplets [9,21,22,42], and the present results do not disagree.

### 4.2. Mother–Young Bonding

The duration of labor increases as the weight of the progeny increases because large fetuses cause dystocia [19], affecting “maternal experience” and therefore, perhaps, mother–young bonding. Importantly, this factor was not a problem in the present study because maternal energy supplementation during the last third of gestation did not influence the duration of labor. This clarifies the interpretation of our observation (multiparous females) since the birth weight of the progeny across the treatments was similar and that, in general, mother–young bonding was positively influenced by maternal energy supplementation during the last third of gestation. Supplemented dams had a greater maternal ability, quickly cleaning their kids, thus increasing mother–young interactions necessary for bonding. Their kids also stood and found the udder more quickly, two factors that favor the survival of the neonate [70]. In late gestation, the circulating concentration of glucose decreases leading to fat mobilization and an increase in the circulating concentration of Non-Esterified Fatty Acids [71]. These observations are consistent with the notion that the last third of gestation is a period of high maternal energy demand [7] that is compounded by a reduction in feed intake as the fetoplacental units grow, especially if there is a large number of fetuses [9]. Therefore, any increase in energy intake in the last third of gestation would be expected to increase glucose supply and promote glucose transfer from the dam and the fetus. The outcome would be an enhanced maternal oxytocin response, thus enhancing maternal behavior [24,72], and an increase in the vigor and therefore survival of the neonate [67,70].

The ability of the neonate to stand and find the udder quickly may be related to an increase in the circulating concentrations of cortisol and glucose [73], leading to an increase in respiration rate that modulates homeothermic mechanisms [55]. Conversely, any delay in the newborn standing would lead to greater loss of heat and energy expenditure, resulting in hypothermia and, ultimately, death [54,70,74]. This interpretation is consistent with the work of Dwyer et al. [75,76] who found that maternal nutritional restriction negatively impacts mother–young bonding by reducing cleaning time and increasing rejection of the neonate. Importantly, in addition to nutritional strategies, genetic strategies could be used to increase the maternal ability and progeny behavior [70,74,77], strengthening the interactions between mother and neonate, and thus mother–young bonding, and reducing neonatal mortality.

In conclusion, maternal energy supplementation during the last third of gestation in multiparous goats did not influence birth weight and had no consistent effects on measures of neonatal wellbeing. However, it improved mother–young bonding and would thus be expected to improve neonatal survival under field conditions. Further research is needed to elucidate the impact of gestational energy supplementation on post-weaning growth and the subsequent reproductive efficiency of the progeny.

## Figures and Tables

**Table 1:** Nutrient composition (Dry Matter basis) of the diets offered to pregnant goats during the last third of gestation, compared with the nutrient requirements for a 50 kg pregnant goat with low physical activity [43].

		Treatment		
	NRC Requirements	T-0	T-150	T-300
Energy supplement g		0	150	300
Alfalfa		ad libitum	ad libitum	ad libitum
ME (Mcal)	4.06	2.68	3.09	3.5
Protein (%)	18.3	13.5	16	18.5
Energy supplementation				
Ingredient composition (% in diet)				
Cracked shelled corn			10.0.	
Bakery waste			35.1	
Cornhusk			22.8	
Soymeal			10.0	
Brewers dry grains			10.0	
Cottonseed			5.0	
Molasses			5.0	
Minerals			2.0	
Nutrient composition of the energy supplement				
ME Mcal/kg			2.75	
CP %			17.0	
NDF %			20.1	
ADF %			7.8	

CP: Crude Protein; NDF: Neutral Detergent Fiber; ADF: Acid Detergent Fiber; ME: Metabolizable energy. NRC: Nutritional Requirements of Small Ruminants [43].

**Table 2:** Birth weight (BWT), rectal temperature (RT), heart rate (HR), and respiratory rate (RR) in the progeny from French Alpine goats that received 0 (T-0), 150 g/head daily (T-150), or 300 g/head daily (T-300) of energy supplement during the last third of gestation.

	BWT (kg)	RT (°C) *	HR (ppm) *	RR (rpm) *
Treatment/*p* value	0.37	0.21	0.23	0.59
T-0 (Control)	3.5 ± 0.6	38.5 ± 0.6	193 ± 19	69 ± 19
T-150	3.7 ± 0.7	38.6 ± 1.8	182 ± 30	74 ± 35
T-300	3.8 ± 0.7	38.0 ± 1.7	191 ± 12	79 ± 20
Birth type/*p* value	0.15	0.04	0.69	0.05
Singleton	3.8 ± 0.8	37.8 ± 1.9 ^b^	193 ± 19	71 ± 22 ^b^
Twin	3.7 ± 0.5	38.4 ± 0.7 ^ab^	187 ± 24	69 ± 22 ^ab^
Triple	3.3 ± 0.7	38.9 ± 1.1 ^a^	190 ± 22	88 ± 35 ^a^
Sex/*p* value	0.02	0.23	0.62	0.98
Female	3.4 ± 0.5 ^b^	38.5 ± 0.6	188 ± 20	72 ± 20
Male	3.8 ± 0.7 ^a^	38.2 ± 1.5	189 ± 26	75 ± 31
Interaction treatment * Birth Type	NS	NS	NS	NS
Interaction treatment * Sex	NS	NS	NS	NS

* Newborn variables were assessed within the first two hours of life. Data of nutritional treatment are presented across sex and birth type of kids. Data of birth type are presented across nutritional treatment and sex of kids. Data of sex of the kids are presented across nutritional treatment and birth type. ^a,ab,b^ Superscripts represent difference among treatment groups within variables (*p* < 0.05).

**Table 3:** Mother–young bonding, assessed within the first hours of life, in French Alpine goats that had received 0 (T-0), 150 g/head daily (T-150), or 300 g/head daily (T-300) of energy supplement during the last third of gestation.

	DL (h)	TC (min)	DFS (sec)	TS (min)	DCU (min)
Treatment/*p* value	0.26	0.001	0.001	0.04	0.32
T-0	1.5 ± 0.2	28.8 ± 6.1 ^ab^	18.8 ± 6.1 ^b^	29.6 ± 7.6 ^ab^	11.8 ± 3.5
T-150	1.6 ± 0.4	25.8 ± 6.6 ^b^	15.9 ± 3.5 ^b^	26.5 ± 4.6 ^b^	11.9 ± 3.1
T-300	1.7 ± 0.3	55.5 ± 18.3 ^a^	29.5 ± 6.8 ^a^	31.1 ± 5.5 ^a^	10.4 ± 3.4

Data of nutritional treatment are presented across sex and birth type of kids. Abbreviations: DL, duration of labor; TC, time taken to clean the newborn; DFS, delay to first suckling; TS, time taken for the newborn to stand; DCU, delay to first connection with the udder. ^a,ab,b^ Superscripts represent difference among treatment groups within variables (*p* < 0.05).

## Data Availability

The data presented in this study are available on request from the corresponding author.

## Data Repository Resources

The data presented in the manuscript is part of the thesis entitled “Effect of diet on the maternal behavior of goats at birth, neonatal vitality and their metabolic profile”, in which the first author of the manuscript is the author of the thesis. The thesis will be submitted to the Universidad Autónoma de San Luis Potosí this year.
